# Cancer Registration in Nepal: Current Status and Way Forward

**DOI:** 10.31729/jnma.4192

**Published:** 2019-04-30

**Authors:** Gambhir Shrestha, Kishore Kumar Pradhananga, Rashmi Mulmi, Krishna Prasad Subedi, Bhola Siwakoti

**Affiliations:** 1 Department of Cancer Prevention, Control and Research, B.P. Koirala Memorial Cancer Hospital, Bharatpur, Chitwan, Nepal

**Keywords:** *cancer*, *national cancer registry program*, *Nepal*

## Abstract

Cancer registration is an organization for the systematic collection, storage, analysis, interpretation and reporting of data on subjects with cancer. Cancer registry was initiated in 1995 and expanded as the National Cancer Registry Program since 2003 by B.P. Koirala Memorial Cancer Hospital with the support of World Health Organization. National cancer registry program currently includes 12 hospital-based registries. First time in Nepal, B.P. Koirala Memorial Cancer Hospital piloted population-based cancer registry in 2013, which included 15 districts covering 25.8% of the total population of Nepal. National cancer registry program is important to assure the quality of data from all the registries to ensure the availability of reliable and valid data of cancer cases. This will further help policymakers to develop preventive and control strategies against cancer. This paper reviews the current status of cancer registries in Nepal and discusses challenges and future perspectives related to national cancer registry program. National cancer registry should further include major hospitals in Nepal to give scientific information on cancer trends by community, provinces and regions and to analyze on the survival of cancer cases.

## INTRODUCTION

Cancer is the second leading cause of deaths worldwide.^1^ With rapid population growth and ageing worldwide and an increase in exposure to risk factors, the incidence of cancer is increasing.^2^ The GLOBOCAN 2018 estimated 18.1 million new cases of cancer and 9.6 million deaths from cancer in 2018.^3^ Around 70% of cancer deaths occur in low and middle-income countries.^4^ Asia only consists of about 48.4% of cancer incidence.^5^ GLOBOCAN 2018 estimates that the age-standardized cancer incidence and mortality rates in Nepal to be 103.7 and 77.8 per 100,000 population in Nepal.^6^ Approximately 30–50% of cancer can be prevented.^7^

Cancer registration is an organization for the systematic collection, storage, analysis, interpretation and reporting of data on subjects with cancer. There are two main types of cancer registry: hospital-based and population-based cancer registries. These registries provide vital information for policymakers to assess cancer burden in the country, plan health services and develop preventive and control strategies. They also provide enough opportunity to conduct research such as time trends, topography patterns and survival analysis evaluating different treatment options in different stages of the disease.^8,9^

## CANCER SCENARIO IN NEPAL

With a change in lifestyle, dietary habits, and high consumption of tobacco and alcohol,^10^ the incidence of cancer is increasing in Nepal. Availability of comprehensive cancer care within the country has made possible for more cancer cases to be diagnosed and treated. Cancer has been recognized as an important disease of public health importance in Nepal. According to the 10 years (2003–2012) consolidated report of the hospital-based cancer registry, female composed of 53.4% and male 46.6% of total new cancer patients.^11^ The most common age group being 50–54 years in female and 60–64 years in the male. Cervical cancer is the topmost cancer followed by breast and lung in the female. Similarly, in male lung cancer was the top most common cancer followed by stomach and colorectal cancer. Majority of cases were from Kathmandu Valley, the capital of Nepal. Similar trend was observed in the consecutive year 2013, 2014 and 2015.^12–14^ Nepal does not have mortality data on cancer. However, WHO had estimated it to be around 6900 male and 7400 female deaths due to cancer in the year 2014.^15^

## CANCER REGISTRATION IN NEPAL

**Figure 1.**
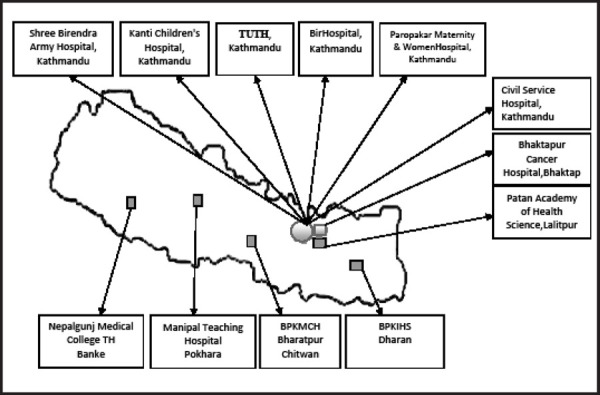
Sites of National Cancer Registry Program in Nepal.

First time in Nepal, B.P. Koirala Memorial Cancer Hospital Management Committee (BPKMCH) in 1995 initiated a Hospital-based Cancer Registry (HBCR) as a pilot program, with objective to assess the cancer load and the types of cancer in three major hospitals in Kathmandu i.e. Bir Hospital, Kanti Children's Hospital and Tribhuvan University Teaching Hospital (TUTH).^11^ In 2003, BPKMCH initiated the National Cancer Registry Program with the support from World Health Organization, which included seven hospitals viz. BPKMCH (Chitwan), Bhaktapur Cancer Hospital (Bhaktapur), Bir Hospital (Kathmandu), Tribhuvan University Teaching Hospital (TUTH) (Kathmandu), Kanti Children's Hospital (Kathmandu), B.P. Koirala Institute of Health Sciences (Dharan) and Manipal Teaching Hospital (Pokhara). These hospitals were selected based on their catchment areas, diagnostic and treatment facilities for cancer. The increasing incidence of cancer and development of diagnostic and cancer treatment facilities in Nepal, National Cancer Registry Program (NCRP) further included five hospitals in 2013 to cover a wide range of cancer patients throughout the country. They are Paropakar Maternity and Women's Hospital (Kathmandu), Patan Academy of Health Sciences (Lalitpur), Civil Service Hospital (Kathmandu), Shree Birendra Army Hospital (Kathmandu) and Nepalgunj Medical College Teaching Hospital (Nepalgunj) (Figure 1).^11^ Currently, 12 hospitals are included in the NCRP; BPKMCH is the main hub to collect, analyze and report data from all the selected hospitals. The number of new cancer cases has been depicted (Table 1). The NCRP was commenced to achieve the following objectives:

1. To assess the burden of cancer stratified by age, gender, residence, topography, morphology and treatment.
2. To undertake epidemiological research.
3. To help assess the quality of hospital care and cancer services.
4. To contribute in active follow up.
5. To plan and develop preventive and control strategies against cancer in the country.

**Table 1:** Number of new cancer cases recorded in NCRP.

Year	1995/96 n (%)	2003 n (%)	2015 n (%)
Male	539 (51.8)	1488 (45.8)	4483 (46.1)
Female	502 (48.2)	1763 (54.2)	5235 (53.9)
Total	1041 (100)	3251 (100)	9718 (100)

## 1. HOSPITAL-BASED CANCER REGISTRY

A Hospital-based Cancer Registry (HBCR) is concerned with the recording of information on the cancer patients seen in a particular hospital.^16^ HBCR have ready and easy access to medical records and collect many clinical data variables. The present HBCR includes 12 major referral hospitals from different parts of the country where most of the diagnosis and treatment of cancer cases are undertaken. Hence, it may reflect the trends and pattern of cancer in Nepal to a significant extent. HBCR can also contribute to monitoring and evaluating the patient care by providing information on the subjects with cancer, the treatment they received and its compliance and the follow-up. However, it cannot provide incidence and mortality of cancer in a defined population because it is not possible to define the catchment populations, that is the populations from which the cancer cases in the data arise.

## 2. DATA COLLECTION

The registry staffs visit medical record section of the selected hospitals to collect the information of cancer patients. The information is then recorded in a standardized form consisting of personal identification number, socio-demographic characteristics, date of diagnosis, basis of diagnosis, tumour site, stage, morphology and treatment. The collected data are entered in excel software. Double/multiple cases are checked rigorously and excluded. All cancers are coded according to the International Classification of Diseases for Oncology (ICD-03).

## 3. POPULATION-BASED CANCER REGISTRY

A Population-based Cancer Registry (PBCR) seeks to collect data on all new cases of cancer occurring in a well-defined population over a given period of time.^16^ That is why PBCR is considered the gold standard to describe the accurate data on cancer burden in a defined population. First time in Nepal, BPKMCH piloted the PBCR in 2013 covering 15 districts with 25.8% of total population of Nepal.^17^ It further plans to identify clusters and extend PBCR with the lesson learnt. Nepal Health Research Council also started PBCR in January 2018 initially covering Kathmandu Valley i.e. Kathmandu, Bhaktapur and Lalitpur districts. There is a need for many institutions working together for gathering information for introducing national PBCR. In our neighbouring country India, NCRP comprised of 26 PBCR and 7 HBCR.^18^

## USES OF CANCER REGISTRY

1. Epidemiological data and research: The cancer registry information can be analyzed to generate incidence rate, cancer site-specific incidence rates stratified by many different variables such as age, gender, residence, site, etc.^8^ These data are of great value for international comparisons. These data are of great value for international comparisons and researches. Prediction of cancer incidence for the future can also be done using various statistics in the cancer registry data. For example, the cancer incidence rate in Nepal for 2020 is predicted to be 38.5 and 41.4 per 100,000 in males and females respectively based on HBCR.^19^
2. Planning and evaluation of cancer control activities: Cancer registry helps to establish public health priorities and forecast future needs by monitoring cancer occurrence in relation to the prevalence of important risk factors. It also helps to assess and monitor the effectiveness of public health interventions such as tobacco control, vaccination against Human Papilloma Virus and screening programs in a community or a defined population.^20^ It also helps to predict future cancer burden and hence help to develop long term programs for cancer control.
3. Survival analysis: Survival analysis can be done from the mortality and follow up data obtained from cancer registry.

## CHALLENGES OF CANCER REGISTRY

1. Cancer coverage: At present, NCRP includes only 12 hospitals. These hospitals are the major hospitals from different parts of Nepal that diagnose and treat cancer. With an increase in advances in technology and human resources, cancer treatment centers are also increasing. Incorporating these centers in the present registry is a major challenge.
2. Timeliness: Another big challenge faced by cancer registry is timeliness of data collection, analysis and reporting.
3. Follow-up and survival data: Mortality data and survival analysis can be estimated from cancer registry; however, it needs to have adequate follow up of each cancer case. Follow up by the cancer registry personnel was impracticable in the past because of inaccessibility of mobile/telephone technology to majority of the people. Nevertheless, at present most of the people have access to mobile/telephone facility which would aid in follow up of the cancer cases. The individual hospital should be involved in active follow up of the cases and provide the data to the main cancer registry hub.
4. Quality of data: The quality of cancer registries depends upon the quality of data and accuracy of the diagnosis.^21^ The data should be accurate and complete. The present registries show incompleteness mainly in the morphology, stage, treatment etc. It has also been difficult to capture outpatient department (OPD) based cancer patients. The missing contact number and follow up further hinder in the survival analysis.
5. Registry personnel and training: Unlike in developed countries, Nepal has limited staffs assigned for cancer registry job. This also has a great impact on the quality of data and reporting of the data. This might be one of the reasons that registries of middle and low-income countries have low quality.^21^ The internationally recognized software for cancer registry Canreg5 should also be oriented regularly to the registry personnel. As the registry staffs are changing in hospitals, this further requires the assignment to other staffs and training for them. Training on statistical analysis and quality of data should also be regularized.
6. Support: Support from and to the institutions to record and analyze the data should be provided. The individual institution should be made capable to use the data for its own plans and improvement of cancer care. NCRP should be supported by the Government and other stakeholders.

## WAY FORWARD

Cancer registries are the best way of obtaining information on the burden and pattern of cancer as well as basis for research on cancer etiology and prevention. Hospital and population based cancer registries are both important and need to be strengthened in the country. Completeness of data of cancer cases and encompassing all cases remain the main challenges of cancer registries. The Goverment of Nepal should play an important role in the systematic registration of cancer cases by supporting national cancer registry program, which will help to develop preventive and control strategies against cancer in the country.

## Conflict of Interest

**None.**
